# Torpid Liver

**Published:** 1890-08

**Authors:** 


### TORPID LIVER.

If the liver is inactive, and consequently fails to make bile enough, the poisonous, waste elements, which should be eliminated in this manner, are retained. The bile is not retained, because it is not made. The materials for the bile are not bile, any more than alkalies and oils are soap. One of the elements of bile is a resinous substance called cholesterine. If this is not carried off properly, very serious and sometimes fatal consequences follow. In the liver itself, it accumulates and forms gall-stones, a diseased condition accompanied with the greatest pain. Gall-stones are sometimes so nearly pure resin that they can be ignited and burned.

One of the symptoms of a torpid liver, is a brassy taste in the mouth, indicating the presence of cholesterine.

Another symptom is specks before the eyes, and these specks are of cholesterine, deposited in the crystalline lens of the eye, where they intercept the rays of light. Sometimes these specks float about, moving with each movement of the eyeball. If these specks become very abundant, they form an impediment to vision.

If the liver is not doing its full duty in the manufacture of bile, the digestive apparatus suffers greatly. A person with a torpid liver is always lean, for he is unable to digest the fat making elements of the food. One with hard, plump tissues cannot possibly have a torpid liver; for a pretty good liver is absolutely necessary to the deposit of a large amount of adipose tissue.

Another consequence of torpidity of the liver is that the food is not well absorbed after it is digested. Such persons may eat enough to be fat, but their food does them no good, beyond maintaining existence.

The gastric juice is a very corrosive fluid, and if the quantity of bile produced is insufficient, the gastric juice is not neutralized as thoroughly as it should be when it meets the food in the small intestines; and as the small intestines have no means of defending themselves from its action, irritation is set up. Such persons will have pain in the bowels, just below the liver, and often complain of a tenderness in that region. The trouble is not in the liver, but in the duodenum. Not infrequently, however, this irritation sets up a catarrh, and the catarrh travels up to the liver, and dams back what little bile is made ; and then the bile must be absorbed into the body, and the skin will not only be dingy, but yellow.

If the bile is scanty, it does not exercise proper antiseptic action, and fermentation sets in before the food is completely digested and ready for absorption. Alcohol and carbonic acid gas are formed, and the bowels become bloated, putrefaction takes place, and offensive gases are formed. Poisonous substances are thus developed, which are absorbed to a greater or less degree ; the breath is tainted, and every tissue and portion of the body and the brain itself, all suffer the poisonous effects. The person may have vertigo, and feel dull and unable to concentrate the mind, with overpowering sleepiness after meals. He is being poisoned by poisons generated within his own alimentary canal.

Yet many people who have torpid livers and indigestion, treat it as a trifling matter. It is really a dreadful thing for one's brain to be so poisoned that it cannot even think properly. The nervous system, as a whole, may be affected, and the disturbance may become so great as to lead to insanity.

The bile is a natural laxative, and stimulates peristaltic action. If the bile is deficient in quantity, then the action of the bowels is partially paralyzed and excretions which should pass off are retained for days and even weeks. During all this time, poisonous substances are generated and being absorbed. It follows that a person with a torpid liver is sick and miserable, and suffers from an innumerable multitude of ills.

If the liver is too torpid to attend to its duty of regulating the supply of sugar, the digested sugar passes directly into the blood, and brings on that disease known as diabetes, which is often very difficult to cure.

Again, the liver may fail to perform its function, and consequently the refuse matters of the body are not completely reduced and changed as they should be to enable them to be thrown off by the organs of elimination. This condition is often made apparent by a whitish, brick-dust, or a pinkish sediment in the urine. These sediments mean that the liver is torpid, and is not converting the waste substances which come to it in the form of uric acid, into urea. Uric acid, or its derivatives, is often deposited around the joints, and the person may have an attack of rheumatism, pleurisy, gout or some allied affection. Nature must do something with this worse than useless material, so she deposits it around the joints, in order to save the delicate membranes of the heart and brain and lungs from suffering from their presence. Sometimes, in place of rheumatism, the person will have neuralgia or a one sided head-ache.

